# Long-term survival with extended lateral lymphadenectomy for lateral lymph node recurrence after laparoscopic abdominoperineal resection for rectal adenosquamous carcinoma: a case report

**DOI:** 10.1186/s40792-018-0440-5

**Published:** 2018-04-10

**Authors:** Takahiro Yokose, Seiichiro Yamamoto, Takeshi Nagase, Toshio Kanai, Kiminori Takano, Taku Fujii, Mai Tsutsui, Motohito Nakagawa, Hiroki Ochiai, Kaori Kameyama

**Affiliations:** 1 Department of Surgery, Hiratsuka City Hospital, 1-19-1, Minamihara, Hiratsuka-shi, Kanagawa 254-0065 Japan; 2 Department of Colorectal Surgery, National Cancer Center Hospital, 5-1-1, Tsukiji, Chuo-ku, Tokyo, 104−0045 Japan; 3 Department of Pathology, Hiratsuka City Hospital, 1-19-1, Minamihara, Hiratsuka-shi, Kanagawa 254-0065 Japan

**Keywords:** Adenosquamous carcinoma of the rectum, Recurrent lateral lymph node tumor, Lateral lymphadenectomy

## Abstract

The patient was a 54-year-old female who presented with the chief complaint of melena. Lower gastrointestinal endoscopy detected a type 1 tumor extending from the anal canal to the rectum. CT did not detect any distant metastasis. Proximal D3 lymphadenectomy with laparoscopic abdominoperineal resection was performed for stage IA rectal cancer. In the histopathological examination, the tumor was identified as stage IIIa adenosquamous carcinoma. Although the patient underwent postoperative adjuvant chemotherapy with S-1, a recurrent left lateral lymph node tumor was detected on CT and PET 12 months later. The patient underwent the treatment with mFOLFOX + bevacizumab for 6 months. However, the tumor continued to progress, and therefore, extended lateral lymphadenectomy was performed 21 months after the first surgery. The patient did not undergo postoperative adjuvant therapy and is alive without recurrence 90 months after the first surgery and 70 months after the reoperation. Adenosquamous carcinoma of the rectum is a rare histological type of colorectal cancer for which there is no effective treatment besides surgical resection, and its prognosis is known to be worse than that of adenocarcinoma. Since there has been no report of long-term survival after extended lateral lymphadenectomy for recurrent lateral lymph node tumors following surgery for adenosquamous carcinoma of the rectum, herein, we report the case with a review of the literature.

## Background

Adenosquamous carcinoma of the colon is defined as a tumor comprised of both adenocarcinoma and squamous cell carcinoma and is a rare histological type of colorectal cancer, with an incidence of about 0.1% of all colorectal cancers [1]. Patients with this type of cancer often present with advanced disease at their first visit to the clinic. Because surgery is the sole established effective treatment for adenosquamous carcinoma, the prognosis of adenosquamous carcinoma remains poor compared with that of adenocarcinoma [2].

Herein, we report a recently encountered case of a patient who survived for a long term after extended lateral lymphadenectomy for recurrent lateral lymph node tumors following surgery for adenosquamous carcinoma of the rectum.

## Case presentation

The patient was female who was 54 years old at the time of her first visit to the clinic. In the previous year’s screening, she had tested positive for fecal occult blood, but the condition remained untreated. As the symptoms of melena gradually developed, she came to the clinic.

### Medical history

Medical history shows cervical cancer after conization (at age 41).

### Hematology findings

No notable abnormalities were found. Tumor markers (CEA 0.8 ng/mL, CA19-9 12.13 U/mL, and SCC 1.5 ng/mL) were within normal ranges.

### Lower gastrointestinal endoscopy

There was a type 1 tumor (25 mm in diameter) extending from the anterior anal canal wall to the lower rectum (Rb).

### Pathological diagnosis of the biopsy specimen

The tumor was diagnosed as invasive poorly differentiated carcinoma with a tendency for cornification and was suspected to be squamous cell carcinoma.

### Thoracoabdominal CT

Contrast-enhanced, irregular thickening of the lower rectal wall was observed. Lymphadenopathy or distant metastasis was absent.

The preoperative diagnosis was cT1b(sm)N0M0 stage IA, and the patient required surgery.

### First surgery

Proximal D3 lymphadenectomy with laparoscopic abdominoperineal resection was performed. Because the preoperative diagnosis was cT1b(sm)N0M0, lateral dissection was not performed.

### Resected specimen

**Fig. 1:**
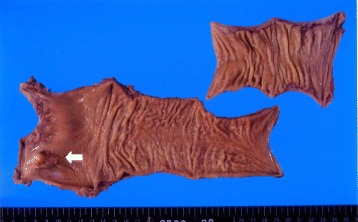
Surgical specimens of the rectum shows type 1 cancer in the lower rectum (arrow)

There was a type 1 tumor, measuring 22 mm in diameter, extending from the lower rectum (Rb) to the anal canal (Fig. 1).

### Histopathological diagnosis

The diagnosis was adenosquamous carcinoma pT3(A) ly2 v1 pN1(2/16) sM0 stage IIIa.

**Fig. 2:**
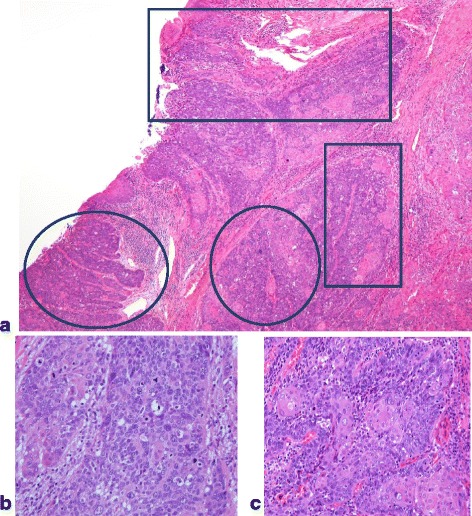
**a** Histological findings of the specimen. Adenocarcinoma component (round line) and squamous cell carcinoma component (square line) are demonstrated. **b** Tumor cells with enlarged nucleus make solid or tubular structure (adenocarcinoma component). **c** In this area, tumor shows apparent keratinization (squamous cell carcinoma component)

The tumor was composed of an approximately 1:1 ratio of adenocarcinoma to squamous cell carcinoma components (Fig. 2a–c).

Lymph node metastasis was only found in #251, which was a result of metastasis of the squamous cell carcinoma component.

### Postoperative course

Due to wound infection on the perineum, incision drainage was performed and the patient was discharged on postoperative day 15.

### Clinical course after the first surgery

**Fig. 3:**
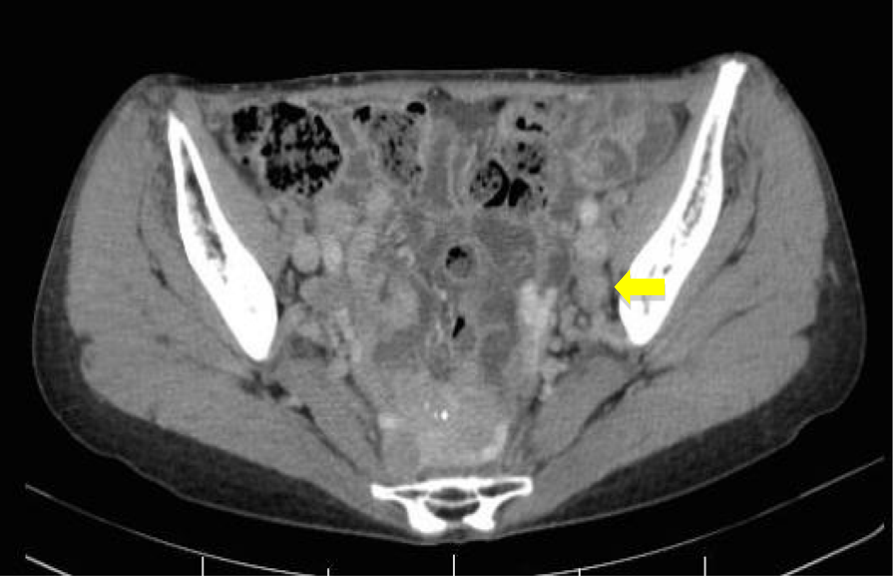
Abdominal CT scan 12 months after surgery showed a lymph node swelling at the left external iliac region (arrow)

**Fig. 4:**
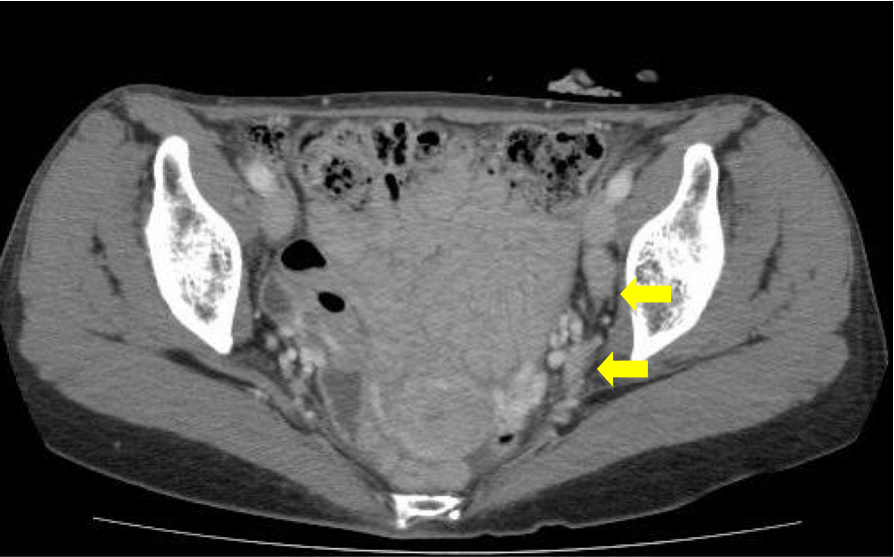
Abdominal CT scan 18 months after surgery showed an enlarged lymph node at the left external and internal iliac regions (arrow)

The patient underwent postoperative adjuvant chemotherapy with S-1 (80 mg/day) for 1 year. On CT, performed 12 months after the first surgery, enlargement of the left lateral and inguinal lymph nodes was detected (Fig. 3). Concomitant PET showed a high concentration of fluorodeoxyglucose in the left lateral lymph nodes, and the patient was diagnosed as having a recurrent tumor of the lateral lymph node. As there was no standard strategy for the treatment of recurrent rectal adenosquamous carcinoma, we expected tumor regression by induction chemotherapy followed by conventional lateral lymph node dissection, and chemotherapy with mFOLFOX6 + bevacizumab was indicated for the next 6 months. On CT, performed 18 months after the first surgery, the tumor exhibited an increased propensity to metastasize to the lateral lymph nodes of the left internal and external iliac regions (Fig. 4), and no distant metastasis was detected. Because the tumor did not respond to the chemotherapy, extended radical surgery was necessary for the curative resection. Therefore, the patient was sent to a referral hospital.

### Reoperation (21 months after the first surgery)

As there was no sign of right lateral lymph node metastasis, unilateral extended left lateral and inguinal lymphadenectomy with combined resection of the external iliac vein, internal iliac artery and vein, obturator nerve, and pelvic nerve plexus was performed.

### Histopathological diagnosis (Fig. 5a–c)

Metastases of the adenosquamous carcinoma were observed in 5 out of 9 of the resected lateral lymph nodes. The tumor was composed of approximately an 8:2 ratio of the squamous cell carcinoma to adenocarcinoma components. There were a number of necrotic cells in the adenocarcinoma component. Therefore, the response to chemotherapy was rated grade 2. However, there were only a few necrotic cells in the squamous cell carcinoma component. Consequently, the response to chemotherapy was rated grade 1a. There were no metastases to the inguinal lymph nodes.

**Fig. 5:**
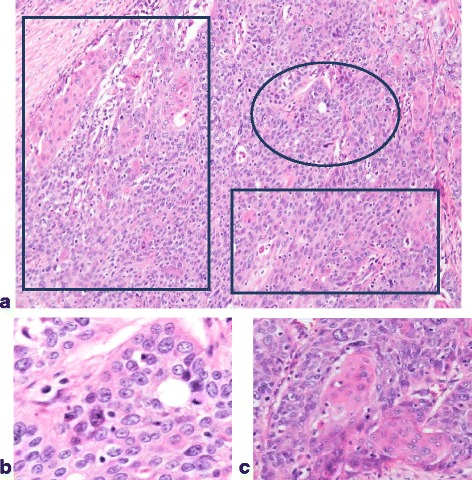
**a** Histological findings of the specimen reveal that the adenocarcinoma component (round line) and the squamous cell carcinoma component (square line) are demonstrated. **b** Tumor cells with enlargements of round nuclei form small ducts and solid pattern (adenocarcinoma component). **c** Tumor cells form eosinophilic nests, indicating keratinization (squamous cell carcinoma component)

The patient did not receive postoperative adjuvant therapy after the reoperation and remains alive without recurrence 90 months after the first surgery and 70 months after the reoperation.

### Discussion

Adenosquamous carcinoma of the colon was first reported by Herxheimer [3] in 1907. According to the eighth edition of the “General rules for clinical and pathological studies on cancer of the colon, rectum and anus” [4], adenosquamous carcinoma of the colon is defined as a tumor comprising adenocarcinoma and squamous cell carcinoma. Although the rules do not mention the proportions of the adenocarcinoma and squamous cell carcinoma components in the tumor in their definition, previous reports have defined adenosquamous carcinoma of the colon as a tumor with no less than 40% [5] of a squamous cell carcinoma component. Because the tumor in our case contained 50% squamous cell carcinoma component, it was diagnosed as an adenosquamous carcinoma.

Adenosquamous carcinoma of the colon is a rare form of cancer with an incidence of about 0.1% of all colorectal malignancies reported in Western countries and in Japan [1, 5, 6]. Yokoi and colleagues [7] have reported that the incidence of adenosquamous carcinoma of the colon is slightly higher in males (1.19:1) and that the tumor is more likely to occur in the right colon compared with adenocarcinoma. Furthermore, adenosquamous carcinoma of the colon is often detected at an advanced clinical stage, in which the tumor is already large and is characterized by a high incidence of lymph node metastasis, compared with adenocarcinoma [7–9]. Besides surgical resection, no effective treatment has been established to treat adenosquamous carcinoma of the colon, which is resistant to chemotherapy. Although there have been reports describing the effectiveness of radiation therapy for local recurrence of adenosquamous carcinoma of the colon following surgery [5, 7], the number of cases has been small. Moreover, adenosquamous carcinoma of the colon is known to be a highly malignant form of cancer with a poor prognosis compared with adenocarcinoma. Nishimura and colleagues [2] reported that the 5-year survival rate was 43.3% among 61 patients with adenosquamous carcinoma of the colon. Since the 5-year survival rate of all colorectal carcinomas is 72.1% [10], the prognosis of patients with adenosquamous carcinoma of the colon is worse than that of patients with other colorectal carcinomas.

The efficacy of surgical resection for liver and lung colorectal metastases has been reported. However, for distant metastases to other sites, the treatment guideline for colorectal cancer indicates that surgical resection is to be considered if the tumor is amenable to complete resection, and if curative resection is difficult, chemotherapy and/or radiation therapy should be considered as the options. Although there have been reports describing the efficacy of surgical resection for recurrent lateral lymph node tumor, there is no established treatment strategy for this cancer. Since the efficacy of chemoradiation therapy for adenosquamous carcinoma has not been established, it is considered necessary to insist on surgical resection when considering the treatment options.

Lymph node metastases of adenosquamous carcinoma of the colon are often a result of metastasis of the adenocarcinoma component. While there have been some reports indicating that the squamous cell carcinoma component is also present in the metastatic lesion, the latter type, composed solely of squamous cell carcinoma, is believed to be rare [5, 8]. In our case, the lymph node metastasis observed during the first surgery was a bona fide squamous cell carcinoma, but the small size of the metastatic lesion made it difficult to make an accurate diagnosis. Therefore, we expected tumor regression by induction chemotherapy followed by conventional lateral lymph node dissection, and chemotherapy with mFOLFOX6 + bevacizumab was indicated. However, in the lymph node metastases resected during the reoperation, the tumor was composed of an 8:2 ratio of squamous cell carcinoma to adenocarcinoma components, demonstrating the dominance of the squamous cell carcinoma component. In consideration of the fact that, in this case, the adenocarcinoma component showed a grade 2 response to chemotherapy, in retrospect, there might have been other treatment options besides surgical intervention, such as combining radiation therapy and/or incorporating combination therapies using drug(s) effective for the treatment of squamous cell carcinoma.

## Conclusion

In Japan, there have been several reports of surgical treatment for adenocarcinoma in recurrent lateral lymph node metastases following surgery. However, there have been no such reports for adenosquamous carcinoma, based on searches of the database in the Japan Medical Abstracts Society or PubMed. Besides surgical resection, there has been no effective treatment for adenosquamous carcinoma of the colon. Therefore, as shown in this case report, surgical resection should be considered as a treatment option for recurrent tumor localized in the lateral lymph nodes.
